# Extracorporeal Shock Wave Lithotripsy for Gallstones: The Shock of the Eighties

**DOI:** 10.1155/1990/48487

**Published:** 1990

**Authors:** A. Darzi, J. R. T. Monson

**Affiliations:** 1 Department of Surgery The Meath Hospital Dublin Ireland; 2 Department of Surgery Mayo Clinic Rochester Minnesota 55905 USA; 3 Academic Surgical Unit St. Mary's Hospital Medical School London W2 1NY United Kingdom


*HPB Surgery*, 1990, Vol. 2, pp. 227–231
Reprints available directly from the publisher
Photocopying permitted by license only
© 1990 Harwood Academic Publishers GmbH
Printed in the United Kingdom

### REVIEW ARTICLE

### EXTRACORPOREAL SHOCK WAVE LITHOTRIPSY FOR GALLSTONES: THE SHOCK OF THE EIGHTIES

A. DARZI
*Department of Surgery, The Meath Hospital, Dublin, Ireland.*

J.R.T. MONSON
*Department of Surgery, Mayo Clinic, Rochester, Minnesota 55905, USA.*

*(Received 6 February 1990)*

KEY WORDS: Bile acids, lithotripsy, gallstones, efficacy-safety

### INTRODUCTION

The application of ESWL for gallstones has developed out of its extremely effective use in the fragmentation of kidney stones[1]. Indeed, ESWL has revolutionized the treatment of nephrolithiasis, dramatically reducing the number of lithiasis-related kidney operations.

Several technologies are being used to produce shock waves under water[2]. The first, developed by Dornier, involves high-voltage underwater discharge of sparks. The sudden evaporation of water results in the generation of shock waves. The shock waves are concentrated by a semiellipsoidal reflector in a focus into which the gallstone is positioned by ultrasonographic guidance. Since the shock waves have to be transmitted through water or through tissue that has a high water content, close contact has to be established between the water medium of the lithotriptor and the patients body. Therefore, either the patient is placed into a water bath (as was the case with first-generation lithotriptors) or the conduction of the shock waves into the body is mediated by a pliable membrane overlying the water medium of the lithotriptor (as is used in the second-generation instruments described below).

In the more recently developed technologies, shock waves are generated either piezoelectrically (the technique used by EDAP and Wolf) or electromagnetically (Siemens). The piezoelectric device consists of many small crystals arranged in a semiellipsoidal dish. Each crystal is electrically activated through a separate generator to produce shock waves under water. The shock waves are, again, bundled into a focus into which the gallstone is positioned. The piezoelectric technique allows a high degree of accuracy and precision in targeting the stone and subsequent delivery of the desired fragmentation energy[3].

Correspondence to: John R.T. Monson, Assistant Director, Academic Surgical Unit, St. Mary's Hospital Medical School, London W2 1NY, U.K.

In the electromagnetic lithotriptor, the shock waves are conducted via a flexible membrane into a closed water container. From there, they are bundled by an acoustical lens into a focus.

All three techniques (spark, piezoelectric, and electromagnetic) have been shown to be effective in fragmenting stones. However, the energy generated by the piezoelectric or electromagnetic devices appears to be somewhat lower than that produced by spark discharge[2]. This explains the need for general or spinal anaesthesia in the studies in which spark technology was used[4,5]. In more recent studies, especially those employing the piezoelectric technique, lithotripsy is being performed with little or no sedation of the patient[6].

If shock waves of sufficiently high-impulse frequency are delivered for a sufficient length of time, cholesterol stones can usually be disintegrated. However, high-intensity treatment, which may achieve successful fragmentation, also increases the incidence of tissue damage, pain and the need for anaesthesia[2]. Thus, the success of fragmentation must be balanced against the risk of tissue damage and the need for anaesthesia. On the other hand, because the piezoelectric machines generate lower energy levels, most patients can be treated without analgesia or sedation. This means that retreatment to achieve complete fragmentation is now a practical solution to greater stone loads.

### PATIENT SELECTION FOR ESWL

The traditional selection criteria for ESWL of gallbladder stones are, with a few notable exceptions, similar to those used for oral dissolution therapy[4,7,8]. As with oral dissolution treatment, ESWL requires the presence of radiolucent gallstones in a functioning gallbladder. The latter is defined as a gallbladder that opacifies during an oral cholecystogram. As is also the case with oral gallstone dissolution therapy, ESWL is not satisfactory if the stone diameter exceeds 3 cm. However, in contrast to oral dissolution therapy, ESWL becomes very ineffective if more than three stones are present[4]. Another important requirement for ESWL treatment has been that the patient must be symptomatic, i.e., he must have experienced biliary pain. However, as experience with ESWL accrues, this criteria will undoubtedly come under serious re-evaluation.

According to the above tight selection criteria, more than 70% of the gallstone population will be considered unsuitable for this form of treatment, and in many cases this is because of their gallstone characteristics. We have, however, shown that it is possible to extend the scope of lithotripsy and dissolution therapy to a wider group of patients[6]. Although this approach will produce a substantial number of failures initially, it allows this treatment to be offered to more patients with gallstone disease, while at the same time learning to identify those patients who are not going to be suitable for the combined treatment.

### CLINICAL STUDIES OF ESWL COMBINED WITH BILE ACID DISSOLUTION THERAPY

Published data on the clinical use of ESWL for gallstone treatment emanate mainly from two medical centres in West Germany[4,9]. The two studies comprised more than 300 patients. Only about 30% of the patients referred to the investigators were found to be eligible for ESWL. Patients were excluded from ESWL for one or more of the following reasons: more than three stones present (45% of referred patients), gallbladder not visualized by oral cholecystography (16%), stones were calcified (15%), stones were too large (10%), and other reasons (14%). With a few exceptions, all of the patients who underwent lithotripsy were also treated with a combination of ursodeoxycholic acid (UDCA) and chenodeoxycholic acid (CDCA). The UDCA-CDCA combination was administered as 7.5 mg/kg/day of each bile acid at bedtime, starting 12 days before lithotripsy and continuing until 3 months after complete disappearance of the stones. The efficacy of the lithotripsy-bile acid combination treatment was greatly influenced by the stone characteristics. The best results were observed in patients with single stones that measured < 30 mm in diameter. In this group, 60% of the stones had disappeared after 3 months and another 35–40% after a total of 12–18 months of postlithotripsy therapy with the UDCA-CDCA combination. The therapeutic success in patients who had two or three stones was less impressive. Within 3 months, 48% of the stones had disappeared, but after a total of 12–18 months of the oral therapy, this figure had increased only to 67%. In contrast, by extending the criteria, we have calculated that we would include up to 60% of our patients who might otherwise have been treated by cholecystectomy. Our stone free rate within two months of the first lithotripsy session is 11 percent. Similary, 24 percent were stone free at 2–4 months, 42 percent at 4–8 months, 64 percent at 8–12 months, and 78 percent at 12–18 months.

To study the influence of the stone characteristics on the result of therapy, we compared patients with a stone profile compatible with the conventional criteria to patients with a stone profile unsuitable according to the conventional criteria. The number of patients clear of gallstones in the group considered suitable according to conventional criteria was significantly higher than the group of patients with a stone profile considered unsuitable at 0–2 months (7/36 vs 3/68, $p < 0.05$), and at 2–4 months (12/36 vs 9/63, $p < 0.05$).

The clearance rates reported by Sackmann *et al.*, are better than those in our experience. However, when comparing the stone free rate of patients with stone profile similar to those employed by the Munich group the results are comparable[2]. In spite of this, we anticipate that the long term future of ESWL will to a large extent depend upon its ability to treat a greater percentage of gallstone sufferers than they suggest.

### SAFETY

Before undertaking clinical studies we determined the safety limits of the EDAP LT–01 system and have found that a dose of 6,000 shock waves per treatment session is both safe and effective in fragmenting gallstones. The experiments detailing both the *in vitro* and animals studies performed have been described in detail elsewhere[10,11]. Overall morbidity with lithotripsy and with dissolution therapy has been low. Piezoelectric lithotripsy appears harmless at the doses we have used and is well tolerated by patients without analgesia or sedation[6]. We have had no mortality and only one patient has developed acute pancreatitis — an episode that resolved spontaneously — in a total experience of over 600 sessions of ESWL.

Repeated treatment at dosages of 6000 shock waves or less has had no untoward effect when treatment sessions are separated by at least a 7 day interval[11]. A few patients, particularly the elderly, experience back discomfort when lying prone, and sometimes this has led to their treatment time being shortened.

### RATIONALE FOR ADJUVANT TREATMENT WITH ESWL

Stone fragmentation with lithotripsy, however, is only the first step, because fragment clearance is the ultimate aim of successful gallstone treatment. Whilst ninety per cent of fragments clear the urinary tract in three months after renal stone lithotripsy, gallbladder stone fragments after lithotripsy appear to require twelve to eighteen months to achieve a 90% clearance[4]. Sackman and colleagues, who reported the initial results with gallstones, used a bile acid combination as their adjuvant therapy. The rationale for this combination was based largely on an *in vitro* study where gallstone dissolution was shown to be significantly accelerated following fragmentation with lithotripsy[12].

This study suggested that the ideal fragment size, after lithotripsy, was 2 mm or less. Although particles of this size can be achieved under optimal conditions *in vitro*, under *in-vivo* conditions, localization of the stones may be less than ideal, and larger fragments may remain in the gallbladder after shock wave application. Whilst it seems logical to treat patients with residual fragments in the gallbladder with bile acids, it adds considerably to the cost of the treatment. Furthermore, it ignores the possible role that mechanical ejection may have on fragment clearance. This ejection would depend on both the size of the fragments in relation to the cystic duct diameter and the contractility of the gallbladder. Contractility itself appears to be impaired in patients with gallstones, although this does not appear to be further impaired by lithotripsy[13]. In order to assess the efficacy of lithotripsy and dissolution therapy, used alone or in combination, we randomised 35 patients to one of three treatment groups: lithotripsy alone, dissolution therapy alone or the combination of lithotripsy and dissolution therapy. All patients had symptomatic gallstones, functioning gallbladders, and the stone profiles were comparable in each group. Lithotripsy was administered using a piezo-electric lithotripter (EDAP LT–01), and dissolution therapy consisted of combined bile acid and terpene administration. Clearance was assessed at six months using both ultrasound and oral cholecystography, and patients with less than 50% clearance at the end of 6 months were considered as failures. The number of patients with total or partial clearance in the combined group (7 out of 10) was significantly greater than those in the lithotripsy alone group (0 out of 10, $p < 0.002$). This study suggests that mechanical ejection alone plays little part in emptying the gallbladder of fragments and that lithotripsy must be combined with dissolution therapy to achieve effective clearance.

### CONCLUSIONS

In summary, it is difficult to assess the exact role for ESWL and dissolution therapy in the future management of patients with gallstones. Patient acceptability is high as the treatment gave little or no discomfort. The costs of treatment in our hands would compare favorably with cholecystectomy and the complications have been few. However, the late costs particularly in terms of stone recurrence and their possible complications are, as yet, unknown. The treatment of gallstones is in a state of flux at present with the addition of other newer procedures such as percutaneous intubation and direct gallstone dissolution[14], percutaneous cholecystolithotomy[15], and laparoscopic cholecystectomy. The future management of patients with gallstones may incorporate many of these techniques adapted to suit specific situations. Our results would suggest that ESWL and dissolution therapy will have a role in the treatment of a substantial number of patients with gallstones.

### *References*

1. Chaussy, C.H., Schmiedt, E., Jocham, D., Brendel, W., Forssmann, B., Walther, V. (1982) First clinical experience with extracorporeal induced destruction of kidney stones by shockwaves. *J. Urol.*, **127**, 417–22
2. Coptcoat, M.J., Miller, R.A.,. Wickham, J.E.A. eds. (1987) Lithotripsy II. London: BDI Publishing
3. Reeders, R.A. (1989) EDAP LT–01. *Journal of Lithotripsy and Stone Disease*, **1**, 237–42
4. Sackmann, M., Delius, M., Sauerbruch, T. *et al.* (1988) Shock Wave Lithotripsy of gallstones. The first 175 patients. *New Eng. J. Med.*, **318**, 393–97
5. Ponchon, T., Barkun, A.N., Purgol, B., Mestas, J.L., Lambert, R. (1989) Gallstone Disappearance after Extracorporeal Lithotripsy and Oral Bile Acid Dissolution *Gastroenterology*, **97**, 457–63
6. Darzi, A., Monson, J.R.T., O'Morain, C., Tanner, W.A., Keane, F.B.V. (1989) Extension of selection criteria for extracorporeal shock wave lithotripsy for gallstones. *Br. Med. J.*, **299**, 302–3
7. Fromm, H. (1986) Gallstone Dissolution Therapy, current status and future prospects. *Gastroenterology*, **91**, 1560–70
8. Magnuson, T.H., Lillemoe, K.D., Pitt, H.A. (1989) How many Americans will be eligible for biliary lithotripsy. *Arch. Surg.*, **124**, 1195–2000
9. Greiner, L., Wenzel, H., Jakobiet, Ch. (1988) Combination of extracorporeal shock-wave fragmentation and bile acid dissolution treatment of gallstone: Further data and experience. *Gastroenterology*, **94**, 1512
10. Darzi, A., Monson, J.R.T., Carey, P.D. *et al.* The influence of shock waves and gallstone characteristics on fragmentation by extracorporeal shock wave lithotripsy (ESWL) — an *in vitro* study. *Surg. Res. Comm.*, 1990; 8: 73–80
11. Darzi, A., Reid, I.M., Kay, E., Monson, J.R.T., Tanner, W.A., Keane, F.B.V. Piezo-electric Lithotripsy and Soft Tissue Injury — Safety limits in the experimental and clinical setting. *Gut*, (in press)
12. Neubrand, M., Sauerbruch, T., Stellaard, F., Paumgartner, G. (1986) *In vitro* cholesterol gallstone dissolution after fragmentation with shock waves. *Digestion*, **34**, 51–9
13. Spengler, U., Sackmann, M., Sauberbruch, T., Holl, J., Paumgartner, G. (1989) Gallbladder motility before and after extracorporeal shock-wave lithotripsy. *Gastroenterology*, **96**, 860–3
14. Thistle, J.L., May, G.T., Bender, C.E., *et al.* (1989) Dissolution of Cholesterol Gallbladder Stones by Methyl Tert-Butyl Ether Administered by Percutaneous Transhepatic Catheter. *New Engl. J. Med.*, **320**, 633–9
15. Kellett, M.J., Wickham, J.E., Russell, R.C.G. (1988) Percutaneous Cholecystolithotomy. *Br. Med. J.*, **296**, 453–5
16. Burhenne, J.H., Stoller, J.L. (1985) Minicholecystostomy and Radiologic Stone Extraction in High Risk Cholelithiasis Patients. *Am. J. Surg.*, **149**, 632–5

*(Accepted by S. Bengmark 6 February 1990)*

